# Quality of Life in Patients Living with Stoma

**DOI:** 10.4314/ejhs.v31i5.11

**Published:** 2021-09

**Authors:** Wuletaw Chane Zewude, Tilahun Derese, Yisihak Suga, Berhanetsehay Teklewold

**Affiliations:** 1 St. Paul's Hospital Millennium Medical College, Department of Surgery, Addis Ababa, Ethiopia; 2 St. Paul's Hospital Millennium Medical College, Department of Surgery, Addis Ababa, Ethiopia; 3 St. Paul's Hospital Millennium Medical College, Department of Surgery, Addis Ababa, Ethiopia; 4 St. Paul's Hospital Millennium Medical College, Department of Surgery, Addis Ababa, Ethiopia

**Keywords:** Colostomy, Ileostomy, Quality of life, QOL-S, Stoma patients

## Abstract

**Background:**

Background and Objective: Quality of life of patients can be affected a treatment. A good quality of life is essential to achieve a goal in treating patients. This study aims to assess stoma related quality of life.

**Methods:**

A cross-sectional study was done at St. Paul's Hospital millennium Medical College from February 1 to July 31, 2019. A structured questionnaire was used to interview patients and review charts of patients to retrieve information on sociodemographic variables, type, and indications of the stoma. Data was collected using structured questionnaire adopted from the City of Hope and Beckman Research Institute after modifications to make it in line with the Ethiopian context.

**Results:**

The mean score for the overall quality of life for stomata was 7.42 ± 0.53. Around 70% of patients have adjusted their dietary style due to stoma. More than half of them reported feelings of depression following stoma surgery. Only 34% of patients resumed sexual activity and only 11% were satisfied with it. None of them were enrolled in stoma association or support group. Factors such as type of ostomy (temporary/permanent), adjustment in dietary style due to stoma, depression, change in diet for not passing gas in public, and change in clothing style had significant effects on overall quality of life and its subscales (*P* < 0.05).

**Conclusions:**

This study demonstrated that living with stoma has a greater impact on the overall aspect of quality of life.

## Introduction

When patients faced with the diagnosis of carcinoma or debilitating chronic disease and the hope of cure, a stoma may seem a small price to pay. Quality is measured by considering physical health, functional status, social, psychological and spiritual wellbeing. Living with a stoma is a challenging situation for various reasons including uncontrolled gas passage through it, odor, diarrhea, and leakage around the stoma or appliance. It would take several months for the patients to adjust to this difficult time. At that point, the patients' QoL becomes paramount for the remaining time.

Ostomy formation is one of the therapeutic procedures performed to manage bowel dysfunction due to various reasons, however it affects the Quality of life (QOL) of patients. World health organization (WHO) defines QOL as an individual's perspective of his/her health status concerning a few aspects- physical, psychological, economic, social and environmental (1).

A stoma can result in a change in body image and influences the physical, mental, emotional, and social life of the patients significantly. A good QOL is essential to achieve a holistic approach in treating patients. A study done in China to assess stoma related QOL using a stoma self-care agency scale and health hope index showed that patients had difficulties in work and social institutions. Additional concerns pointed out were sexuality, body image and stoma itself (2).

A long-term effect on the quality of life of members of the United Ostomy Association of America after 5 years of ostomy surgery was assessed using a questionnaire. Their report has shown that patients feel better as they live longer with the stoma (3). A research done on Iranian by ostomy society has shown that factors such as the type of ostomy, the underlying disease that had led to the stoma formation, depression after ostomy, dissatisfaction with sexual activities, problem with the location of ostomy and change in clothing style affected the Quality of Life (4). Even if stoma surgeries are among the most common procedures in Ethiopia, studies assessing the impacts of the stoma on quality of life on these patients are lacking (5). Ostomies can lead to intensified distress and suffering of patients as a result of skin irritation (76%), pouch leakage (62%), offensive odor (59%), reduction in pleasurable activities (54%), and depression/anxiety (53%) (6). In such circumstances, it is worthwhile to assess the quality of life in the evaluation of the outcomes of various therapeutic procedures along with their final impact on patients' lives. (7) Quality of care and training provided to patients can be associated with their subsequent QoL. A study done in one of the biggest hospitals in the country has shown that only half of the nurses among those who are responsible to prove ostomy care have good knowledge and skill to give care and training to patients (8).

The main aim of this study is to assess the quality of life of patients living with an ostomy. The impact of ostomy on the quality of life of patients can be related to physical wellbeing, psychological wellbeing, social wellbeing, and spiritual wellbeing. (9–24).

## Methods and Patients

A facility-based prospective cross-sectional study was conducted at SPHMMC, a teaching hospital in Addis Ababa, Ethiopia from February 1 to July 31, 2019. All patients who were living with stoma and were having follow up at the surgical referral clinic in SPHMMC during the study period were included in the study, except those who are critically ill, ongoing adjuvant chemo/radiotherapy, disabling mental illness, significant comorbidity that can affect QOL, complicated stoma at time of interview and having disabling symptomatology from trauma. We used a structured questionnaire that is prepared by assembling the result of available works of literature and adopting from previous studies and modified in line with the objectives of the study and our country/community cultural and social status. It is used after reviewed and approved by the college's ethical review board. The assessment tool we used is adopted from the City of Hope Quality of Life -Ostomy Questionnaire (COH-QOL-Ostomy). We choose this tool because it is a standard QoL assessment tool as well as it has been used in low and middle-income countries, which have reasonably the same socioeconomic status as Ethiopia has. The tool has two sections. The first section includes 42 questions about demographics, disease, treatment, ostomy specifics and other personal characteristics such as diet, work, and activities. The second section contains 43 items. These items are further divided into four subscales: physical wellbeing, psychological well-being, social wellbeing and spiritual wellbeing. The highest and lowest points imply the highest and lowest level of QOL, respectively. Adding all the scores of each subscale and dividing their sum by the number of items in that subscale is used to calculate the total score of questions in each subscale. A total QOL score is obtained adding the scores on all 10-point items and dividing by the total number of items.

Data were collected and entered, categorized, coded, and summarized using Epi-Info 7.0 and was exported to SPSS version 23 for further analysis. The analysis was verified using descriptive interpretation. Frequency distributions of demographic variables were obtained and the distribution of quantitative variables described with the mean, median, and standard deviation. To test the difference between the two groups, Pearson's Chi-square test was applied for binary and qualitative variables. For quantitative variables, t-test and ANOVA were used. Univariate analysis was employed to show the association between the dependent and independent variables and multivariate regressions were used to identify predictors of QOL. Differences were considered significant at *p < 0.05.*

## Results

In total, 39 males (60.9%) and 25 females (39.1%) participated in the study. The mean age of the patients was 49.3 ± 17.5 years. The mean age of male and female patients was not significantly different (51.8 ± 17.3 vs. 45.4 ± 17.2, *P* = 0.153). The majority of study participants (39, 60.9%) were residents of urban and semi-urban areas, while 25 of them (39.1%) reside in rural areas of the country. Colostomy was the most common type of stoma surgery (*n* = 60, 93.8%) and the rest are ileostomy. Permanent ostomy was done in 59.4% of patients (n = 38). Stomas were made for 67.2% (*n* = 43) of the patients due to cancers. According to the patients' reports, 70.3%, and 64.1% of stoma patients had been forced to change their diet, and clothing style, respectively. Post ostomy creation, 58% of respondents had feelings of depression while assessing their psychosocial concern, but it was only patient who had suicidal thought. Problems related to clothing due to the location of ostomy formed were reported by 59.4% (*n* = 38) of patients. Those patients were forced to change their clothing style.

The majority of patients (78.1%) reported being sexually active before stoma surgery. Only 34% of them resumed sexual activity after the surgery. Among those who resumed sexual activity, two patients reported satisfaction with their current sexual activity and 28.2% of male patients had erectile dysfunction since stoma surgery was done for them.

None of the study participants were members of an ostomy-specific support group or association. Only two of the study participants have access to proper stoma bags while the rest of them use local plastics made for another purpose. The mean length of time for patients to complete ostomy care was 45 (SD ± 6.45) minutes every day.

According to the reports of most patients, the average time required by patients to be familiar and comfortable on self-stoma care was 3 months (SD±1.08). The majority of ostomy patients (73.3%) had no restrictions on the consumption of specific foods such as dairy products, fruits, snacks, and vegetables.

The mean score for the overall QOL in stoma patients was 7.42 ± 0.53. The best outcomes were found for the spiritual subscale, which means, patients have relatively less problems related to their spiritual activities after stoma formation where as their social interaction was affected much as they score lowest in this subscale than the other QOL parameters. (Table 1).

**Table 1:** Mean and standard deviation of COH-QOL-Ostomy subscale scores

QOL Subscales	Mean	Median	Min/Max	SD
Physical QOL	7.3406	7.3000	5.50/8.10	.58790
Psychological QOL	7.4719	7.4000	5.00/8.50	.60065
Social QOL	7.1063	7.0000	5.60/8.20	.51513
Spiritual QOL	7.7781	7.8000	5.50/8.80	.51513
Overall QOL	7.4242	7.5000	5.60/8.50	.53205

Depression is found to be more common in female(75%) patients living with stoma than males (46%)(p<0.05). Depression(56%), change in clothing style due to the stoma(56%) and change in dietary style for not passing flatus in public(56%) were more common among patients living in rural areas than those who are urban inhabitants (p<0.05)(Table 2).

**Table 2:** Comparison between gender, place of residence, time of stoma and indication for stoma with feelings of depression, change in clothing style and dietary change not to pass flatus in public

Variable		Depressed of stoma	P value	Change in clothing style	P value	Dietary change not to pass flatus in public	P value
Gender	Male	46% (18 of 39)	0.018	46% (18 of 39)	0.421	46% (18 of 39)	0.421
	Female	75% (19 of 25)		52% (13 of 25)		52% (13 of 25)	
Place of residence	Urban	41% (16 of 39)	0.001	28% (11 of 39)	0.000	28% (11 of 39)	0.000
	Rural	84% (21 of 25)		80% (20 of 25)		80% (20 of 25)	
Stoma time	Permanent	26 of 38	0.034	21 of 38	0.043	21 of 43	0.570
	Temporary	11 of 26		10 of 26		10 of 21	
Preop diagnosis	Cancer	26 of 43	0.048	21 of 43	0.043	21 of 43	0.043
	Obstruction	9 of 16		9 of 16		9 of 16	
	Trauma	2 of 5		1 of 5		1 of 5	

Independent t test and ANOVA tests have revealed that feelings of depression, change in clothing style, dietary habit change and time to return of appetite were significantly different among the indication of stoma (Common among those patients who are having stoma for malignant disease than those who have benign disease) and type of stoma (more among patients who have permanent colostomy than those who have temporary) (p<0.05).

Univariate analysis indicated that factors such as the type of ostomy (temporary/permanent), adjustment in dietary style due to stoma, depression, change in diet for not passing gas in public, and change in clothing style had significant effects on overall QOL and its subscales (P<0.05) (***which factor has significant impact on which subscale is shown in the table***) (Table 3).

**Table 3:** Mean difference of COH-QOL-Ostomy subscale scores according to demographic and clinical characteristic

Variables		Physical Mean (SD)	Psychological Mean(SD)	Social Mean (SD)	Overall Mean (SD)
Gender	Male(39)	7.4385(.57244)	7.6308(.58000)	7.2154(.51989)	7.4923(.55979)
	Female(25)	7.1880(.59042)	7.2240(.55624)	6.9360(.46805)	7.1800(.43012)
Type of ostomy	Ileostomy(4)	7.3500(.52599)	7.7750(.63966)	7.3750(.34034)	7.6250(.47871)
	Colostomy(60)	7.3400(.59580)	7.4517(.59816)	7.0883(.52176)	7.3533(.53472)
Type of ostomy	Permanent(38)	6.9789(.41730)*	7.0816(.37116)*	6.7447(.27870)*	7.0316(.29964)*
	Temporary(26)	7.8692(.35187)	8.0423(.36788)	7.6346(.25447)	7.8654(.38879)
Cause of ostomy	Cancer (43)	7.0744(.47564)	7.1721(.44149)	6.8070(.32543)*	7.0977(.34330)
	Obstruction (16)	7.8563(.41468)	8.1188(.37098)	7.7062(.17308)	7.9375(.40311)
	Trauma (5)	7.9800(.29496)	7.9800(.39623)	7.7600(.13416)	7.9000(.41833)
Place of residence	Rural (36)	7.2240(.59601)	7.3720(.57700)	7.0360(.46715)	7.2160(.50388)
	Urban (28)	7.4154(.57790)	7.5359(.61408)	7.1513(.54477)	7.4692(.53220)
Change in job after stoma	No change (59)	7.3712(.59191)	7.5051(.61376)	7.1305(.52003)	7.4017(.54282)
	Changed (5)	6.9800(.43243)	7.0800(.10954)	6.8200(.38341)	7.0000(.00000)
Change diet for not passing flatus in public	No (0)	7.9286(.31645)	8.0905(.30644)	7.6238(.27551)	7.9048(.33982)
	Yes (64)	7.0535(.46206)*	7.1698(.46009)*	6.8535(.40317)*	7.1093(.39630)*
Depression	No (2)	7.7815(.45996)*	7.9407(.47252)*	7.5185(.40860)*	7.7222(.48701)*
	Yes (62)	7.0189(.44711)	7.1297(.43003)	6.8054(.35115)	7.1135(.40426)
Adjust in diet due to stoma	No (0)	7.9421(.33051)	8.1368(.28326)	7.6053(.28181)	7.9474(.32892)
	Yes (64)	7.0867(.47749)*	7.1911(.46064)*	6.8956(.44105)	7.1267(.39566)*
Change in clothing	No (2)	7.9304(.29609)	8.0870(.33208)	7.6435(.26939)	7.8696(.34435)
	Yes (62)	7.0098(.42884)*	7.1268(.40989)*	6.8049(.34493)*	7.0902(.39611)*
Problem with location of ostomy	No (4)	7.8115(.37345)	7.9269(.46952)	7.5154(.38956)	7.7692(.40573)
	Yes (60)	7.0184(.48147)*	7.1605(.46935)	6.8263(.38882)*	7.0974(.42711)

* Mean difference is significant and p value is less than 0.05

The results of regression analysis have shown that only type of stoma and adjustments in diet were statistically significant in predicting patients physical, psychological and the overall quality of life scales whereas indication for ostomy and change in clothing style were effective in predicting the social subscale (P<0.05) (Table 4).

**Table 4:** Predictors of overall quality of life and its subscales in the linear regression

Variables	Physical		Psychological		Social		Spiritual		Overall QOL	
	
	SE (β)	β	SE(β)	Β	SE(β)	Β	SE(β)	Β	SE(β)	β
**Gender**	.15	-.01(.614)	.14	-.19(.412)	.10	-.10(.650)	.14	-.16(.088)	.09	-.14(.359)
**Age**	.01	.14(.421)	.01	.11(.996)	.00	.09(.366)	.01	-.37(.868)	.00	.04(.152)
**Stoma type**	.09	.73*(.007)	.09	.79*(.033)	.10	.62*(.021)	.14	.35*(.030)	.08	.75*(.050)
**Indication**	.21	.06(.271)	.14	.08(.162)	.08	.29*(.032)	.14	-.01(.319)	.12	.19(.217)
**Change in** **diet**	.48	.26(.376)	.44	.15(.392)	.33	.02(.582)	.45	.62(.300)	.25	-.01(.552)
**Change in job**	.27	.09(.710)			.18	-.09(.898)	.25	-.18(.714)	.23	-.17(.552)
**Sex** **satisfaction**	.12	-.33(.075)	.10	-.13(.680)	.08	.09(.301)	.05	-.20*(.050)	.10	.07(.510)
**Depression**	.21	.06(.186)	.19	-.07(.887)	.14	-.06(.511)	.19	-.19(.208)	.17	.24(.652)
**Avoid** **beverages**	.25	-.02(.943)	.22	-.06(.300)	.17	-.08(.786)	.13	-.38*(.035)	.21	-.19(.657)
**Problem with** **location of** **stoma**	.19	-.11(.358)	.18	.07(.201)	.13	.06(.024)	.18	.05(.894)	.15	-.22(.620)
**Change in** **clothing**	.27	-.45*(.035)	.34	-.14(.639)	.19	-.42*(.024)	.35	.15(.065)	.25	.14(.355)
**Adjustment** **in diet**	.19	-.37*(.003)	.19	-.35*(.001)	.14	.06(.366)	.21	.12(.086)	.18	-.31*(.001)

* Significant at P <0.05

## Discussion

Our patients have reported worst outcomes on the social subscale of the QoL which is comparable to the reports by Roshini and et al, who have studied a group of 40 patients with stoma in a tertiary care hospital of South India and found that their most intense concerns were stoma related problems such as skin rash with irritation and leakage, difficulties in social and family interactions (14). In this study none of the patients are part of stoma support group and only 2 of them had access to a proper stoma appliance (colostomy or ileostomy bag) while the rest were using a locally available plastic bags produced for other purpose, which is significantly associated with their daily comfort in stoma care unlike reports made by Deger and et al in Turkey, which has reported as their patients are discharged with stoma bags free of charge (12). Use of these plastic bags, which are not designed to be used for stoma care can worsen stoma related local complications because of issues related to poor placement of bags and associated leakage to the surrounding skin (1, 5, 11, 12, 13, 14).

Though many of physicians and nurses involved in ostomy care suggest that patients with stoma should be able to continue wearing their own clothing with slight changes if needed, change in clothing style due to stoma surgery was among major problems for patients participated in this stud (10, 13). This was consistent with studies done across the world. (1, 5, 14, 28) The reasons can be the location of ostomy, weight changes, and changes in body appearance.

The usual advice to patients with stoma by professionals is to return to their normal preoperative diet (10, 13). However, majority of our patients (70%) were forced to change their dietary style and have fear not pass flatus in public. They also need at least 3 months to be familiar with self-stoma care, which can have an effect on their time to return to their routine preoperative work. This has been also shown by two Iranian studies (5, 10), that majority of patients with stoma have been obliged to change their dietary style. Shorter hospital stays contribute for patients to have inadequate information about their dietary style after ostomy surgery. This is also aggravated by the lack of availability of dieticians and the need for reiteration of information once at home. All those things affect how people view their condition in relation to diet after ostomy is formed for them as a treatment. Giving adequate and ongoing advice on a diet by a trained dietician can be effective to these patients to minimize the psychosocial discomfort they experience (10). Those psychosocial burdens related to dietary style are also reflected in our patients involved in this study, which could have been avoided by adequate patient education and ongoing support.

One of the most significantly affected domains in QoL of patients undergoing stoma surgery is the social subscale. This surgery will change the body image of the patient with a huge impact on his/her social interaction and it will sound more if we bring this issue to the community with high social interaction where interdependence is a daily routine like ours, especially among those living in rural and semi urban Ethiopia (29). Participants of a study by Roshini and et al have expressed a preference to avoid any family gatherings because of fear of offensive gas emission, fullness and smell. Significant numbers of them were awkward to be with outsiders and all of them felt that they were a burden to their family and the least score in their QoL assessment was scored in social sub scale (14). Similarly, the results our current study showed that the social subscale scored the lowest among the other QOL subscales. It is perhaps because it is culturally unacceptable to pass a flatus in public among our society, to which an ostomy has already lost a control, which will have a great impact on person's confidence, which will finally reduce their social relations. Another reason to a significantly low social sub scale score in our study may be explained by absence of stoma support groups and ostomy associations as well as access to proper stoma appliance. These factors go hand in hand and lead to some degree of social isolation and this was consistent with other studies (5, 10, and 14).

Sexuality among patients with stoma is a frequently reported concern. In men, the concern may be impotence and infertility, while in women dyspareunia from decreased lubrication may worry them. However pre-operative libido and an interested partner are probably the two most important factors in re-establishing sexual intimacy after the operation (5, 10, 22, 27). In a study done in Turkey by Deger and et al some patients did not answer sexuality/body image questions. The authors attributed this to Turkish socio-cultural life. As some people still believe it is a shame to talk about sexuality in Turkey and have strict taboos (12). Though as of Turkish socio-cultural life, it is shameful to talk about sexuality in majority of Ethiopians, it was not a problem in our current study probably because most of our study participants were urban and semi urban inhabitants and all have answered the sexuality related questions. In a study conducted by Gemmill and et al (25), it has been shown that 70% of patients had sexual activity before stoma surgery, while only 55% of patients resumed their sexual activity after surgery. In this study, the number of people who resumed their sexual activity after surgery is far less than (34%) the number reported in the Gemmill study (25). The findings of the current study are consistent with other studies (5, 10, 15, 20, 22, 27). This may be due to a lack of proper training on sexuality issues to stoma patients in Ethiopia. Therefore, it may be useful to refer stoma patients for counselling and training about sexual health.

Like other studies (5, 14, 15, 26), more than half of the patients in this study were suffering from depression following stoma surgery. Despite the high prevalence of depression in our study subjects, only one of the participants was having suicidal thought and this looks better in contrast to a study by Roshini and et al done in a tertiary hospital of South India where the prevalence of suicidal thought was 20% (14). Since the depression had not been evaluated before stoma surgery, the relevant results should be interpreted cautiously.

Mumtaz Ahmed Khan and et al examined quality of life of patients living with stoma among Muslim Pakistanis. The best quality of life score was to the spiritual (religious) domain of QoL and the major reason mentioned was effective psychosocial counselling by religious leaders (23). This survey as well showed, the spiritual sub scale of our patients quality of life was the most preserved one and the reason mentioned for Pakistani patients may work, because we have relatively comparable spiritual way of life regarding spiritual practices, where almost all our population has a religion to follow and the relation with religious leaders that patients can have. (29)

In conclusion, the findings of this study showed that living with stoma influences the overall aspect of QOL. Proper placement of the stoma can decrease the patients' difficulties. Pre-and post-operative education for the patients and their families is important so that the stoma patients' QOL can be improved. Sexual disorders and feeling of depression were major problems of stoma patients. Sexual and psychological counselling may improve patients' QOL. Access to formal stoma bag is a major problem to this study participants and increasing availability of stoma bag and increasing awareness of patients on stoma bag use may improve quality of life of these patients.
